# The miR-532-E2F1 feedback loop contributes to gastric cancer progression

**DOI:** 10.1038/s41419-022-04832-7

**Published:** 2022-04-19

**Authors:** Shanting Gao, Xiaomin Bu, Yongyue Gao, Zengtao Bao, Wenchao Shi, Lipeng Luan, Huiyu Chen, Baoming Zhang, Qingshui Tian, Wenxian Guan, Liuqing Yang

**Affiliations:** 1 Department of Gastrointestinal Surgery, The First People’s Hospital of Lianyungang, The First Affiliated Hospital of Kangda College of Nanjing Medical University, Lianyungang, Jiangsu China; 2 Department of Clinical Laboratory, Cancer Hospital of the University of Chinese Academy of Sciences (Zhejiang Cancer Hospital), Institute of Basic Medicine and Cancer (IBMC), Chinese Academy of Sciences, Hangzhou, Zhejiang China; 3 Department of Neurosurgery, Nanjing Drum Tower Hospital, The Affiliated Hospital Nanjing University Medicine School, Nanjing, China; 4 Department of General Surgery, Nanjing Drum Tower Hospital, The Affiliated Hospital of Nanjing University Medical School, Nanjing, Jiangsu China; 5 Department of Infectious Diseases, The First People’s Hospital of Lianyungang, The First Affiliated Hospital of Kangda College of Nanjing Medical University, Lianyungang, Jiangsu China

**Keywords:** Gastric cancer, Apoptosis, miRNAs

## Abstract

Gastric cancer (GC) ranks fourth in incidence and mortality worldwide, ascertaining the pathogenesis of GC is crucial for its treatment. E2F1, which regulates the transcription of genes encoding proteins involved in DNA repair, DNA replication, mitosis and survival of cancer patients, functions as a key regulator in GC progression. However, the underneath mechanism of these processes is not fully elucidated. Here, TCGA database analysis, microarray immunohistochemical technique and western blot showed that E2F1 was highly upregulated in clinical GC tissues and correlated with tumor malignancy. In vitro and in vivo assays confirmed the oncogenic function of E2F1. MiR-532 was decreased and negatively correlated with E2F1 in GC tissues. MiR-532 directly targeted and inhibited E2F1 expression, leading to the decrease of ASK1 and elevation of TXNIP, and affected proliferation, cell cycle, apoptosis and DNA damage in vitro and tumor growth in vivo. Moreover, E2F1 serves as a transcriptional repressor to suppress miR-532 expression and a double-negative feedback loop was formed between them. This study demonstrates the significant roles of the E2F1-miR-532 double-negative feedback loop in GC progression and may represent a potential target for GC therapy.

## Introduction

GC is the fourth most commonly diagnosed malignancy globally and the fourth leading cause of cancer death [1]. Fully clarifying the mechanisms behind the initiation and progression of GC and exploring more effective therapeutic approaches are urgently needed.

Being part of E2F gene family, E2F1 acts as a transcription factor and involves in controlling cell proliferation, cell cycle progression, apoptosis, autophagy, DNA replication, DNA damage and repair, development and differentiation [2–7]. In view of its rich functionality, E2F1 plays complicated roles in the initiation and development of malignant tumors [2]. In general, E2F1 serve as a tumor promoter in various tumors via activating the expression of oncogenes, for example, melanoma [8], lung cancer [9], liver cancer [10], renal cancer [11], colorectal cancer [12] and so on. Abnormal expression of E2F1 is common in malignancy that are associated with poor patient survival prognosis, such as bladder cancer, non-small cell lung cancer, prostate cancer and melanoma [13–18]. Therefore, E2F1 serves as a key regulatory factor to affect tumor progression and an attractive target for cancer treatment. Despite recent elucidation of biological functions, however, the regulation of E2F1 expression is poorly understood. Further study of the underlying mechanisms of E2F1 regulation is critical to better understanding of GC pathogenesis.

To elucidate the molecular issues involved in E2F1-related tumor progression, a promising approach is to investigate dysregulated microRNAs (miRNAs). MiRNAs regulate gene expression by combining with the 3’-untranslated regions (UTRs) of target mRNA, resulting in translation inhibition or mRNA degradation [19]. MiRNAs play vital roles in a variety of different cellular processes, for example, carcinogenesis [20]. As different studies hint toward the interaction of E2F1 and oncogenic miRNAs [8, 11, 21–24], an involvement of miRNAs in E2F1-induced GC progression is conceivable. However, the studies related to post-transcriptional regulation of E2F1 expression during GC progression is still rare.

In this study, we showed that E2F1 was specifically targeted by miR-532, which controlled the progression of GC both in vitro and in vivo. Because E2F1 is a transcriptional factor, we investigated crosstalk between miR-532 and E2F1 and demonstrated that E2F1 inhibits miR-532 expression through specific E2F1-binding motifs. Thus, E2F1 and miR-532 form a double-negative feedback loop that contributes to gastric cancer progression.

## Methods

### Patients and tissue samples

21 pairs of fresh patient tissues of GC were obtained from consenting patients who underwent surgical resection at The First People’s Hospital of Lianyungang. The experiments were authorized by the Ethics Committee of The First People’s Hospital of Lianyungang. A signed consent form was obtained from each donor. Samples were frozen in liquid nitrogen after surgeries and conserved at -80 °C. The clinical features of patients are listed in Table 1. Tissue microarray chips containing a total of 100 pairs of GC samples and matched normal adjacent tissues (NAT) were obtained from Shanghai Biochip Co., Ltd (Shanghai, China). According to the staining intensities, the samples were scored as 0-3 [0(negative), 1(weak), 2(moderate), 3(strong)]. The expression of E2F1 was determined as high when IHC score >2 and low when the IHC score <2, respectively. The follow-up data of these GC patients were collected for survival analyses.

**Table 1:** Clinical features of 21 GC patients.

Case	Age	Gender	Pathological Stage
#1	67	male	III
#2	65	male	I~II
#3	58	male	III
#4	49	male	I
#5	71	male	III
#6	70	male	III
#7	55	male	IVA
#8	62	male	II
#9	49	male	II
#10	69	male	III
#11	44	female	II
#12	51	male	II
#13	58	female	II~III
#14	50	male	II
#15	57	male	III
#16	52	male	III
#17	57	female	I
#18	43	female	II
#19	48	male	II
#20	56	male	I
#21	67	male	II

### Cell culture

Human GC cell lines (MKN-45 and AGS) were obtained from the Shanghai Institute of Cell Biology, Chinese Academy of Sciences (Shanghai, China) and recently authenticated (STR profiling) and tested for mycoplasma contamination. Both cells were cultured in DMEM supplemented with 10% fetal bovine serum (Gibco) at 37 °C with 5% CO_2_.

### Transient transfection

MiRNA mimics, inhibitors, negative controls and E2F1-specific siRNAs were synthesized by GenePharma (Shanghai, China). E2F1 overexpression plasmids were synthesized by GenScript (Nanjing, China). An empty plasmid served as the negative control. Lipofectamine 2000 (Invitrogen, Carlsbad, CA, USA) was used for transfection following the manufacturer’s instructions as previously described [23].

### Western blot

Cellular protein was extracted as described previously [23]. Antibodies against E2F1 (ab218527, Abcam, Cambridge, MA, USA), TXNIP (ab188865), ASK1 (sc-5294, Santa Cruz, CA, USA) and GAPDH (sc-25778) were used. GAPDH served as the internal control. ImageJ v1.50e was used to quantify the band intensity.

### Real-time quantitative PCR

Total RNA extraction, reverse transcription and real-time PCR were performed as previously described [11]. The primer sequences were as follows: E2F1 (sense): CAGAGCAGATGGTTATGG; E2F1 (antisense): CTGAAAGTTCTCCGAAGA; CLCN5 (sense): AAGTGGACCCTTGTCATCAA; CLCN5 (antisense): ACAAGATGTTCCCACAG; GAPDH (sense): CGAGCCACATCGCTCAGACA; and GAPDH (antisense): GTGGTGAAGACGCCAGTGGA. The detection of pre-miR-532 levels was performed as previously described [25]. The sequences of the primers were as follows: pre-miR-532 (sense): CCTCCCACACCCAAGGCTTGCA; pre-miR-532 (antisense): CAACGGTCCTACACTCAAGG; U6 (sense): CTCGCTTCGGCAGCACA; U6 (antisense): AACGCTTCACGAATTTGCGT. The relative amount of miRNA was normalized to U6.

### Pull-down assay

For pull-down assay, two DNA probes complementary to E2F1 mRNA and labelled with biotin at the 3’-terminal, was synthesized. A scrambled biotinylated probe was used as negative control (Genescript, Nanjing, China). The probes (8 pmol/ul) were incubated with streptavidin-coated magnetic beads (New England BioLabs, USA) at 25 °C for 1 h to generate probe-coated magnetic beads. Then the MKN-45 lysate was incubated with probe-coated beads at 37 °C for 3 h, with constant rotation. After incubation, beads were washed and treated with Trizol reagent to extract RNA. The sequence of E2F1 probes were as follows: probe 1: TCCTGGGTCAACCCCTCAAG, probe 2: GACAACAGCGGTTCTTGCTC. The extracted RNA was analyzed by qRT-PCR.

### Luciferase assay

The p-MIR-REPORT plasmids were designed to contain the 3′-UTRs of human E2F1 by GenScript (Nanjing, China). MKN-45 and AGS cells were seeded into 24-well plates and co-transfected with E2F1-WT plasmids, E2F1-mut plasmids, miR-532 mimics, inhibitors or negative controls by Lipofectamine 2000. After 48 h of transfection, the cell lysates were collected and the Dual-Luciferase Reporter Assay System (Promega) was used to detect the firefly and renilla luciferase activities [26].

Moreover, to check the direct binding between E2F1 and miR-532 promoter, two synthetic 300 bp DNA fragments (Invitrogen), which include Site 2-WT (include site 2 binding sequence) and Site 3-WT (include site 3 binding sequence), were inserted into the promoter region of pGL3 basic (Ambion), and the insertion was confirmed by sequencing. To test the binding specificity, the sequences that interacted with E2F1 were mutated (all binding positions were mutated), and the mutant two DNA fragments were inserted into the promoter region of pGL3 basic plasmid too. MKN-45 cells were cultured in 24-well plates and each well was co-transfected with firefly luciferase reporter plasmid, β-gal expression plasmid (Ambion) and E2F1 siRNAs using Lipofectamine 2000 (Invitrogen). The β-gal plasmid was used as a transfection.

### Cell proliferation assay

The proliferation rate of GC cells were measured by CCK-8 and EdU assays according to protocols as described previously [27]. In brief, GC cells was seeded in 6-well plates and treatmented with different transfections, 24 h later, cells were collected and reseeded in 48-well plates for EdU assays and 96-well plates for CCK-8, respectively. Cell Counting Kit-8 (Dojindo, Japan) and EdU assay kit (RiBoBio, China) were used to detect the proliferation rates of cells according to the instructions [27].

### Cell cycle and apoptosis assays

To analyze cell-cycle, GC cells were seeded in 6-well plates and treatmented with different transfections, when the density reaches 90%, cells were collected and fixed in 70% ice-cold ethanol overnight. Then the cells were treatmented with RNase (100 μg/ml) and stained with propidium iodide (50 μg/ml) and further analyzed by flow cytometer.

### Chromatin immunoprecipitation

ChIP assays were performed using ChIP assay kit (Millipore, Massachusetts, USA) according to the manufacturer’s instructions. Soluble chromatin was prepared from MKN-45 cells and incubated with an anti-E2F1 antibody (Abcam, UK) or human IgG (negative control). The primers were as follows: for site 1, forward: CAGTCTCATTCTGTCGCCCA, reverse: GCCTGGCCAACATGAAGAAA; site 2, forward: GGGTTTCATCATGTTGGCCA, reverse: GGAAAGGTTTGATTGTTTGCTTG; site 3, forward: AGGCCGGAATAACACACAGA, reverse: ATGTCCTCCAGATGCATGTG; site 4, forward: TCCCAGTTCTACCACGTACT, reverse: ATACACAGGCACAGAAAGGTT.

### In vivo studies

6-week-old male nude mice were purchased from the GemPharmatech (Nanjing, China). All procedures complied with the ARRIVE guidelines and the National Institutes of Health guide for the care and use of Laboratory animals and the guidelines of The First People’s Hospital of Lianyungang. Animals were numbered and randomly divided into groups according to a random number. All the investigators were blinded to group allocation during data collection and analysis. To explore the effects of the E2F1-miR-532 axis on tumorgenesis in vivo, 1×10^6^ MKN-45 cells overexpressing or knocking down E2F1 or miR-532 or control cells were resuspended in 200 μL ice-cold PBS and injected into the armpits of mice, respectively. Volumes were measured approximately every 5 days, at day 25, mice were sacrificed. The sample sizes chosen for assays as previously described [28].

### Statistical analysis

All western blot images are representative of at least three independent experiments. Quantitative RT-PCR, luciferase reporter assay, chromatin immunoprecipitation, cell function assay were performed in triplicate, and each experiment was repeated several times. No data was excluded from the analyses. All statistical tests were performed under the open-source statistics package R or using GraphPad Prism software 7 (San Diego, CA). Data are presented as means ± SEMs. Normality and equal variances among groups were assessed using the Shapiro–Wilk test and Brown–Forsythe tests, respectively. To determine the significance between two groups, an unpaired t-test was performed. ANOVA was used to analyze the differences between more than two groups. *P* values < 0.05 were considered statistically significant.

## Results

### Deregulation of E2F1 in GC samples

**Fig. 1:**
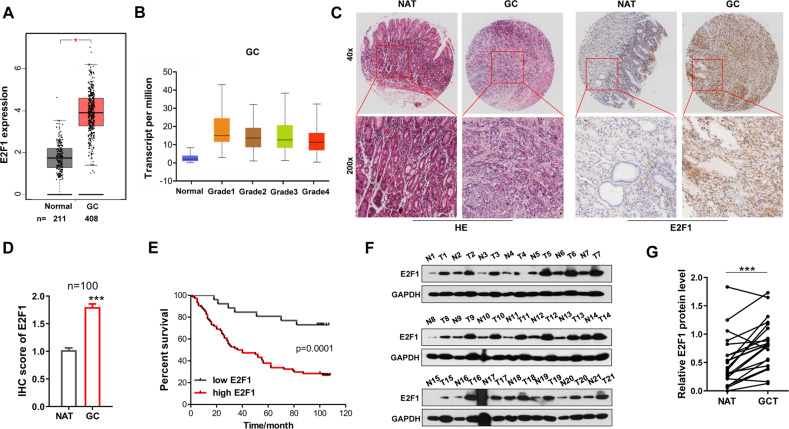
E2F1 deregulation in human GC tissues is associated with poor prognosis. **A** E2F1 expression analysis in 211 normal tissues and 408 GC tissues from TCGA database. **B** E2F1 expression was correlated with the clinical grade of GC in TCGA dataset. **C** Representative images of HE staining and E2F1 IHC staining in GC tissues and matched normal adjacent tissue (NAT) (*n* = 100). **D** Total IHC score of E2F1 in GC tissues and matched NATs (*n* = 100). **E** Kaplan–Meier analysis of survival of patients with GC stratified by E2F1 expression. **F**, **G** Western blot analysis of E2F1 protein levels in another 21 paired GC tumor tissues (T) and normal adjacent tissues (N) samples. **F**: Representative images; **G**: quantitative analysis. *P < 0.05; *** *P* < 0.001.

To demonstrate the clinical significance of E2F1 expression in GC, we firstly compared the E2F1 expression between cancer patients and normal tissues in TCGA database. The results showed that E2F1 mRNA level was greatly upregulated in GC (Fig. 1A). Moreover, data from TCGA database showed that GC malignancy grade rose with increasing E2F1 expression levels (Fig. 1B). Then we measured E2F1 expression in GC tissue specimens from a cohort of 100 GC patients via immunohistochemistry (IHC) staining. We found that E2F1 was significantly upregulated in 92/100 (92%) GC cases (Fig. 1C, D). Notably, GC patients with high levels of E2F1 had significantly shorter overall survival time (Fig. 1E). We further verified the observation by measuring E2F1 levels in another 21 pairs of fresh human GC specimens via western blotting. Compared with the NATs, E2F1 expression levels were greatly upregulated in GC tissues (Fig. 1F, G). The results demonstrate that E2F1 is up-regulated in GC and associates with tumor malignancy and poor prognosis, suggesting that E2F1 may play a key role in GC tumorigenesis.

### E2F1 promotes proliferation and G1/S transition, suppresses GC cells apoptosis and DNA damage in vitro and in vivo

**Fig. 2:**
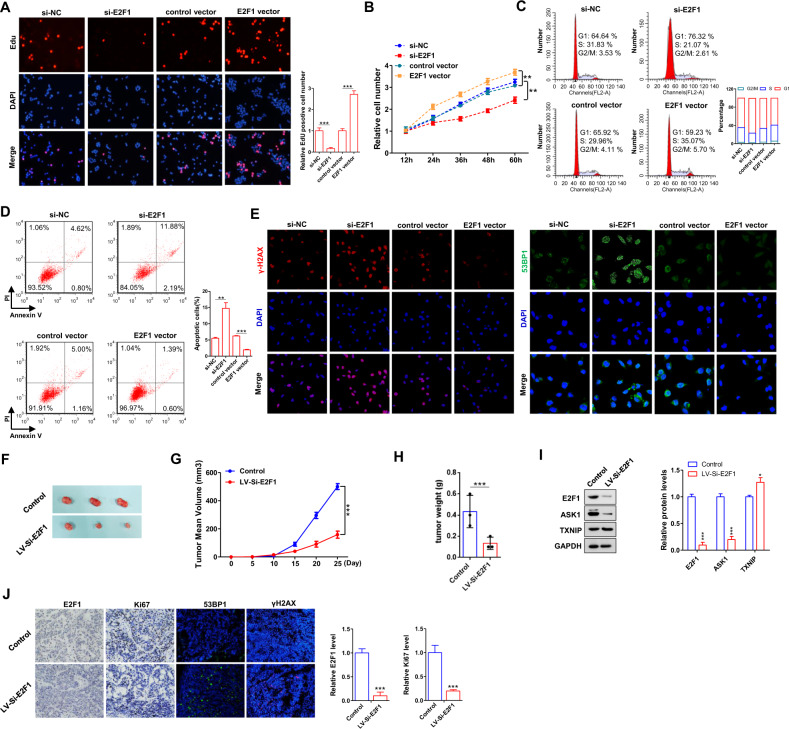
E2F1 functions as an oncogene in GC. **A**, **B** Cell proliferation assays (EdU and CCK8) were performed in MKN-45 cells transfected with si-E2F1 or E2F1 vector. **C**, **D** Representative images and histogram statistics from cell cycle and apoptosis. **E** Immunofluorescence staining of DNA damage markers, γH2AX and 53BP1, in MKN-45 cells transfected with si-E2F1 or E2F1 vector. **F** Representative images of tumors from mice implanted with control MKN-45 cells and E2F1-inhibiting MKN-45 cells. The cells were implanted subcutaneously into 6-week-old SCID mice (3 mice per group). **G** The time course of tumor growth. Tumor volume was measured every 5 days for 25 days after the inoculation. **H** The tumor weights of two groups. **I** Western blotting analysis of E2F1, ASK1 and TXNIP protein levels in tumors from the implanted mice. **J** E2F1, Ki67 and 53BP1, γH2AX staining of tumor sections obtained from two groups. All data are shown as the mean ± S.E. of three separate experiments. *P < 0.05; **P < 0.01; *** P < 0.001.

To further study the function of E2F1 in GC progression, we examined the effects of E2F1 on proliferation, cell cycle, apoptosis and DNA damage in GC cell line MKN-45. E2F1-specific siRNAs (si-E2F1-1, si-E2F1-2 and si-E2F1-3) were used to silence E2F1 expression and si-E2F1-2 showed the strongest inhibitory effect (Supplementary Fig. 1A, B). Western blotting showed that E2F1 knockdown result in markedly decreased ASK1 protein level and increased TXNIP protein level (Supplementary Fig. 1C), the downstream genes of E2F1 [8, 29]. EdU assays, staining to detect nucleotide analogue incorporation into replicated DNA, showed that interference of E2F1 expression significantly reduce the cell proliferation rate (Fig. 2A). Similar results were observed in CCK-8 assay (Fig. 2B). Next, we performed flow cytometry experiments to investigate the effects of E2F1 on cell-cycle progression or apoptosis. Compared with control cells, E2F1 inhibition led to an increase of G1-phase population, and a concomitant decrease of cell number at the S phase and G2/M phase (Fig. 2C). Apoptosis assays revealed that E2F1 knockdown result in increased cell apoptosis (Fig. 2D). Moreover, we determined the effect of E2F1 on DNA damage. Immunofluorescence showed that interference of E2F1 expression substantially increased the accumulation of H2AX histone protein (γH2AX) and 53BP1, markers of DNA double-strand breaks (DSB) [30–32], compared to transfection with si-NC (Fig. 2E). Conversely, overexpressing E2F1 markedly increased ASK1 protein level and decreased TXNIP protein level (Supplementary Fig. 1C). The measurement of functional parameters revealed that E2F1 overexpression promote GC cell proliferation, G1/S transition and suppress cell apoptosis and DSB accumulation (Fig. 2A–E). Therefore, the above results further confirmed that E2F1 promote GC proliferation, G1/S transition and suppress apoptosis and DNA damage of GC cells in vitro.

To further study the effects of E2F1 on GC tumorigenesis in vivo, MKN-45 cells were infected with si-E2F1 lentivirus to suppress E2F1 (Supplementary Fig. 2A, B), and then the cells were implanted to nude mice to establish a xenograft mice model. Consistent with in vitro results, E2F1 knockdown significantly delayed GC tumor growth (Fig. 2F–H). Moreover, tumors from E2F1 silencing group showed decreased E2F1, ASK1 and increased TXNIP levels than control group (Fig. 2I). H&E staining showed decreased mitosis in E2F1 inhibition group compared with the control group, while E2F1 and Ki-67 staining revealed less E2F1 protein level and decreased proliferative activity (Fig. 2J). These results demonstrated that E2F1 promoted GC tumorigenesis in vivo.

### Prediction of E2F1 as a target of miR-532

**Fig. 3:**
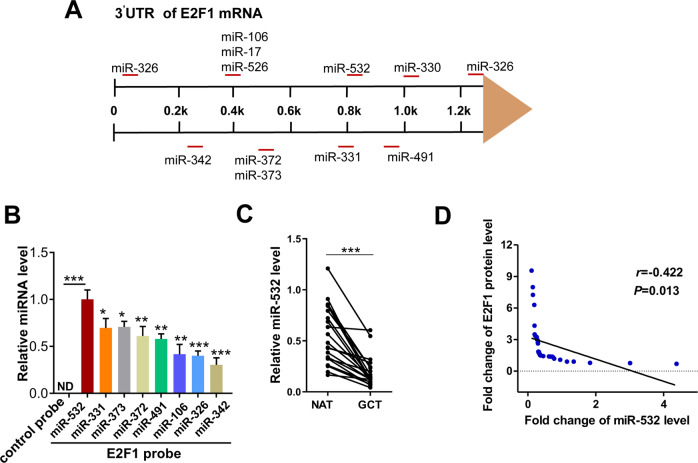
Prediction of E2F1 as a target of miR-532. **A** Schematic overview regarding 12 candidate miRNAs targeting E2F1 3′-UTR. **B** QRT-PCR analysis of miRNA levels in MKN-45 after pulling down by E2F1 mRNA probe or control probe. **C** Representative expression of miR-532 in GC and NAT analyzed by QRT-PCR. **D** Pearson’s correlation scatter plot of the fold change of miR-532 and E2F1 in 21 pairs of GC tissues. *P < 0.05; **P < 0.01; ***P < 0.001.

MiRNAs play critical roles in tumor progression by post-transcriptionally regulating target gene expression [33]. To identify the potential miRNAs targeting E2F1 in GC, candidates were selected by the intersection of bioinformatics software Targetscan, miRWalk, miRanda [28, 34, 35]. Figure 3A showed the candidate miRNAs targeting E2F1 3′-UTR. Then we designed a biotinylated anti-E2F1 mRNA probe and transfected the E2F1 mRNA probe into MKN-45. Forty-eight h later, we pulled down the biotinylated anti-E2F1 mRNA probe by using streptavidin-coated magnetic beads and measured the coprecipitated miRNAs. Of them, miR-532 displayed the highest level (Fig. 3B). Then, the correlation between miR-532 and E2F1 in GC tissues was analyzed. QRT-PCR showed that miR-532 was consistently decreased in 21 GC tissues (Fig. 3C). Furthermore, we used Pearson’s correlation scatter plots to illustrate the inverse correlation between miR-532 and E2F1 protein in GC tissues (Fig. 3D). Combined with the above results, we speculated that miR-532 may potentially regulate E2F1.

### MiR-532 directly regulates E2F1 expression at the posttranscriptional level

**Fig. 4:**
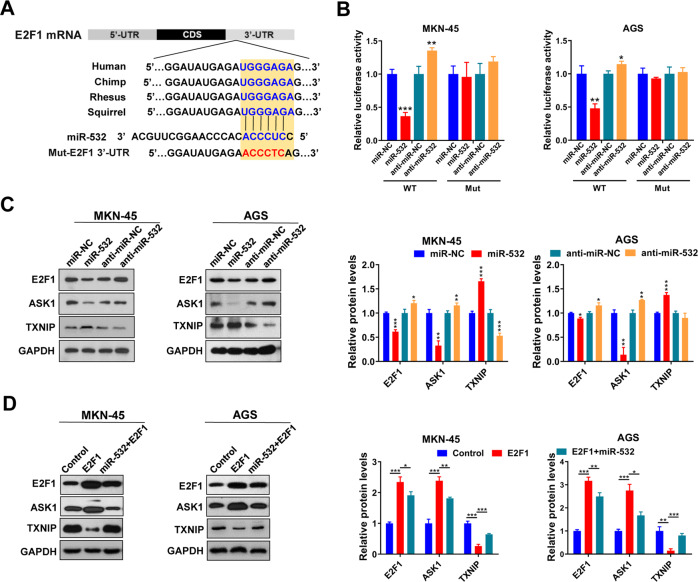
miR-532 can directly inhibit E2F1 by binding to its 3’-UTR. **A** Schematic diagrams of the predicted interaction between miR-532 and E2F1. **B** Relative luciferase activities in MKN-45 and AGS cells transfected with miR-532 mimics or inhibitors. **C** Western blotting analysis of E2F1 protein levels, as well as its downstream protein, ASK1 and TXNIP, in MKN-45 and AGS cells transfected with miR-532 mimics or inhibitors. **D** Western blotting analysis of E2F1, ASK1 and TXNIP protein levels in MKN-45 and AGS cells transfected with E2F1 overexpressing vector or miR-532 mimics plus E2F1 overexpressing vector. *P < 0.05; **P < 0.01; ***P < 0.001.

To further verify that miR-532 suppressed E2F1 expression through the direct interaction with the binding sites in E2F1 3′-UTR (schematic depicting the hypothetical duplexes formed by interactions between the binding sites in the E2F1 3′-UTR (top) and miR-532 (bottom) was showed in Fig. 4A), we constructed a firefly reporter plasmid containing a fragment of E2F1 3′-UTR across the miR-532 binding sites. Then the resulting plasmid was transfected into MKN-45 and AGS cells along with miR-532 mimics, miR-532 inhibitors or negative control RNAs, respectively. As anticipated, overexpressing miR-532 significantly reduced the luciferase reporter activity compared to cells transfected with miR-NC, whereas miR-532 inhibition resulted in an increase in reporter activity compared to cells transfected with anti-miR-NC (Fig. 4B). Then we introduced point mutations into the sites that are complementary to miR-532 within E2F1 3ʹ-UTR (Fig. 4A). As shown in Fig. 4B, when the binding site was mutated, miR-532 overexpression or knockdown lead to a loss of luciferase inhibition.

Next, we measured the effects of miR-532 on E2F1 protein expression levels in MKN-45 and AGS cells transfected with miR-532 mimic or inhibitor, respectively (Fig. 4C, Supplementary Fig. 3A, B). As expected, miR-532 overexpression significantly inhibited E2F1 and ASK1 protein and increased TXNIP protein levels, whereas inhibition of miR-532 increased E2F1 and ASK1 levels and inhibited TXNIP protein (Fig. 4C). In order to further explore whether miR-532 can attenuate the promoting effects of E2F1 on ASK1 and the inhibitory effects on TXNIP, we performed rescue experiments by transfecting E2F1 overexpressing vector or miR-532 mimics plus E2F1 vector into GC cells. As shown in Fig. 4D, miR-532 overexpression markedly counteracted the promoting effects of E2F1 on ASK1 and the inhibitory effects on TXNIP. Taken together, the above results determined that miR-532 specifically regulates E2F1 protein expression at post-transcriptional level.

### MiR-532 inhibits GC cell proliferation, G1/S transition and promotes apoptosis and DNA damage by suppressing E2F1 in vitro

**Fig. 5:**
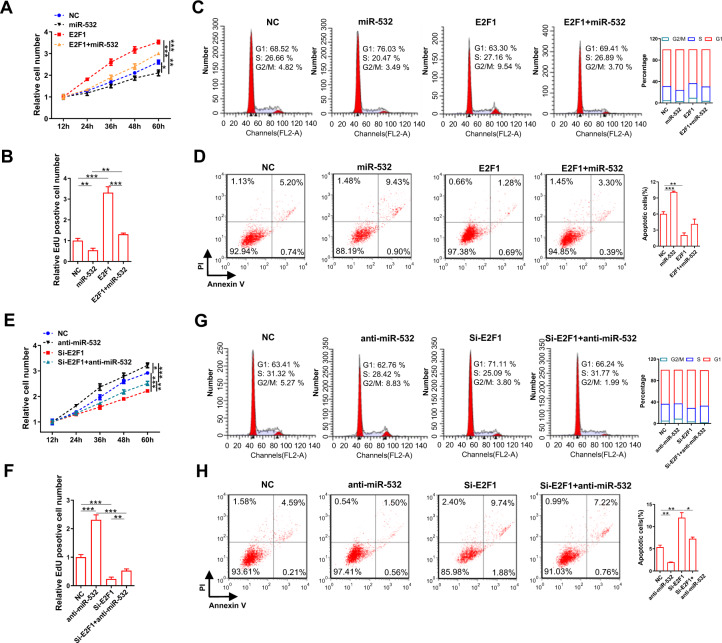
miR-532 inhibits GC cell proliferation, G1/S transition and promotes apoptosis and DNA damage by suppressing E2F1 in vitro. **A, B** Cell proliferation assays (CCK8 and EdU) were performed in MKN-45 cells transfected with miR-532 mimics, E2F1 vector or E2F1 vector plus miR-532 mimics. **A**: CCK-8 assay; **B**: EdU assay. **C, D** Histogram statistics from cell cycle and apoptosis assays. **E**–**H** Analysis of cell proliferation, cycle and apoptosis in MKN-45 cells transfected with miR-532 inhibitors, si-E2F1 or si-E2F1 plus miR-532 inhibitors. *P < 0.05; **P < 0.01; ***P < 0.001.

To further study the biological effects of miR-532 on GC cells, function analyses were performed in MKN-45 cells. Firstly, EdU assays were conducted to investigate the effects of miR-532 on GC cell proliferation. MKN-45 cells transfected with miR-532 mimics exhibited slower proliferation, in contrast, miR-532 inhibition showed the opposite effect on cell proliferation (Fig. 5A, B, E, F; Supplementary Fig. 4). Next, flow cytometry experiments revealed that miR-532 overexpression increase G1-phase population and decrease S and G2/M phase population and promote GC cell apoptosis, whereas miR-532 knockdown showed converse effects (Fig. 5C, D, G, H).

To further determine whether the effects of miR-532 on GC cell function is derived from miR-532-mediated E2F1 suppression, we performed recovery experiments. Functional restoration assays showed that E2F1 significantly reversed miR-532-led inhibition of GC cell proliferation, G1/S transition and miR-532-led promotion of GC cell apoptosis and DNA damage (Fig. 5A–D and Supplementary Fig. 5A). Meanwhile, cells co-transfected with miR-532 inhibitor and E2F1 siRNA showed higher proliferation rates, G1/S transition and lower cell apoptosis and DNA damage recognition foci accumulation compared to cells transfected with E2F1 siRNA alone (Fig. 5E–H and Supplementary Fig. 5B). Altogether, these data strongly show that miR-532 inhibits gastric cancer cell proliferation, G1/S transition and promotes apoptosis and DNA damage by directly targeting E2F1.

### miR-532 and E2F1 form a double-negative feedback loop

**Fig. 6:**
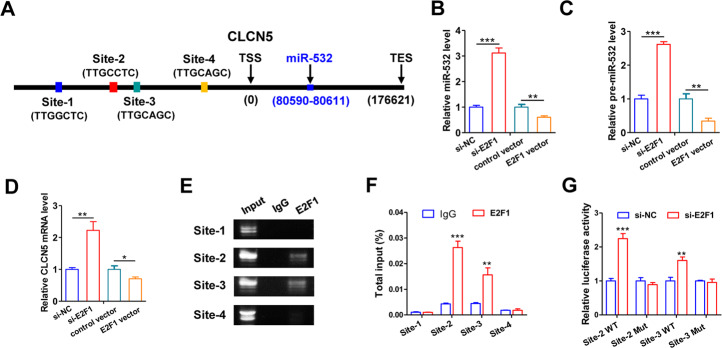
miR-532 and E2F1 form a feedback loop. **A** Schematic illustrating the putative E2F1-binding sites in miR-532 promoter. **B**, **C** qRT-PCR analysis of miR-532 or pre-miR-532 levels in MKN-45 cells after changing E2F1 expression. **D** qRT-PCR analysis of CLCN5 mRNA levels in MKN-45 cells after changing E2F1 expression.**(E**, **F** Direct binding of E2F1 to miR-532 promoter as indicated by ChIP assays. Binding was confirmed by semi-quantitative PCR, followed by gel electrophoresis. **G** Luciferase reporter assay confirmed the suppression of miR-532 promoter by E2F1. *P < 0.05; **P < 0.01; ***P < 0.001.

The mechanism behind the ectopic expression of miR-532 during GC tumorigenesis is largely unclear. We therefore predicted the potential transcription factor could directly regulate the expression of miR-532. Interestingly, bioinformatics analysis identified four E2F1-binding motifs [36] within the promoter region of the CLCN5 gene (miR-532 is located in the third intron of CLCN5 gene) (Fig. 6A). Hence, we explore whether E2F1, a sequence-specific transcriptional factor, could bind to the E2F1-responsive elements and regulate miR-532 expression. Furthermore, we knocked down or overexpressed E2F1 in MKN-45 cells and checked the response of miR-532 by quantitative RT-PCR. Downregulation of E2F1 in MKN-45 cells resulted in a three-fold increase in miR-532 expression compared with cells transfected with a control siRNA, whereas E2F1 overexpression lead to a two-fold decrease in miR-532 expression (Fig. 6B, C). Similar alteration in the levels of the CLCN5 mRNA was observed (Fig. 6D), suggesting that miR-532 alteration was likely due to the transcriptional changes. To check whether E2F1 directly binds specific motifs in miR-532 (CLCN5) promoters, we performed chromatin immunoprecipitation (ChIP) assays in MKN-45 cells. The ChIP assay results confirmed that E2F1 proteins were recruited to binding sites 2 and 3 in the CLCN5 promoter (Fig. 6E, F). Next, we cloned the sequences of E2F1-binding sites 2 or 3 into an upstream region of a firefly luciferase reporter gene and transfected the resulting plasmids into MKN-45 cells. Luciferase reporter assays revealed that E2F1 knockdown increased the transcription of firefly luciferase in both plasmids with E2F1 binding site 2 or 3 sequences; however, when the binding sequences of sites 2 and 3 were mutated, firefly luciferase activity was unaffected by E2F1 inhibition (Fig. 6G). Altogether, these results demonstrate that E2F1 negatively regulates the transcription of miR-532 via specific E2F1-binding motifs in the promoter region.

### The effect of E2F1-miR-532 loop on GC tumor growth in vivo

**Fig. 7:**
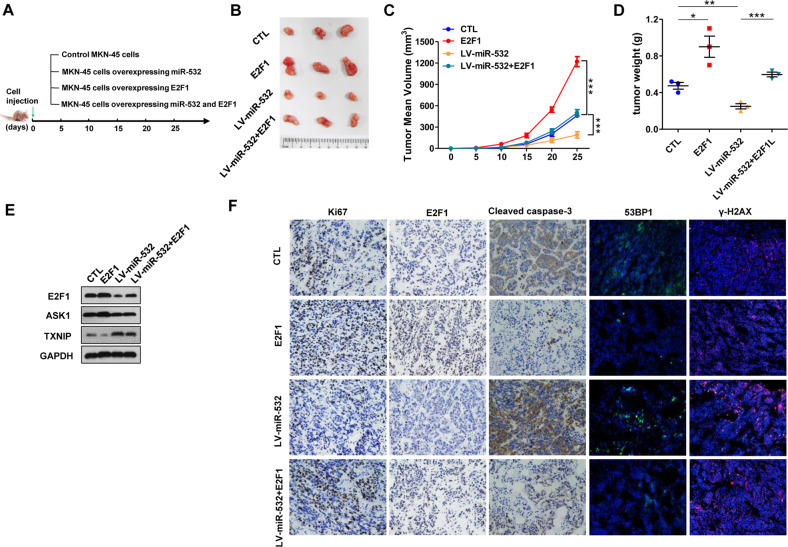
The effect of E2F1-miR-532 loop on GC tumor growth in vivo. **A** A schematic diagram illustrating the experimental design. MKN-45 cells overexpressing miR-532, E2F1 or miR-532 plus E2F1 were implanted subcutaneously into nude mice (3 mice per group) and tumor growth was evaluated on day 25 after implantation. **B** Images of tumors from GC mice. **C** Tumor volume curves. **D** Tumour weights. **E** Western blot analysis of E2F1, ASK1 and TXNIP levels in GC xenografted tumors. **F** IHC staining for Ki-67, E2F1 and Cleaved caspase-3 and IF staining for 53BP1 and γ-H2AX in tumors from four groups. *P < 0.05; **P < 0.01; ***P < 0.001.

To verify the effects of E2F1-miR-532 double-negative feedback loop on GC tumorigenesis in vivo,

MKN-45 cell was infected with miR-532 lentiviral vectors. Efficient overexpression of miR-532 and inhibition of E2F1 by lentiviral vectors is shown in Supplementary Fig. 6. Then we implanted the MKN-45 cells overexpressing miR-532 and/or E2F1 into the armpits of 6-week old nude mice to construct a xenograft model for GC (Fig. 7A). Tumor growth was measured after cell implantation. On day 25, the mice were sacrificed and tumors were removed and weighed. As shown in Fig. 7B–D, a significant increase in the sizes and weights of tumors was observed in the E2F1 overexpressing group compared with the control group, whereas the tumors from miR-532-overexpressing group grew dramatically slower. Restoration of E2F1 weakened the tumor-inhibitory effect of miR-532. Subsequently, total RNA and protein were extracted from tumors and analyzed. As shown in Fig. 7E, tumors from E2F1 overexpressing group showed higher E2F1, ASK1 and lower TXNIP than the control group. However, TXNIP protein levels were elevated, E2F1 and ASK1 protein levels were markedly decreased in LV-miR-532-infected group compared with the control group. Moreover, E2F1 overexpression effectively restored the E2F1, ASK1 protein level decreased by miR-532 and the TXNIP protein level promoted by miR-532 (Fig. 7E).

Then, IHC staining showed less E2F1, lower percentage of proliferative cells and higher apoptosis marker cleaved-caspase 3 expression in LV-miR-532 infected tumors, whereas tumors overexpressing E2F1 showed more proliferative cells and less cleaved-caspase 3 expression than the control group. Restoring E2F1 increased the proliferation rate suppressed by LV-miR-532 and decreased the apoptosis rate promoted by LV-miR-532. Furthermore, we examined changes in the expression levels of key DNA damage-related markers in tumors of each group. IF staining experiments showed that overexpressing miR-532 promoted the expression of γH2AX and 53BP1, co-expression of E2F1 with miR-532 could weaken the level of DNA damage (Fig. 7F). In summary, these results showed the tumor-suppressive role of miR-532 in vivo functioning by targeting E2F1.

## Discussion

**Fig. 8:**
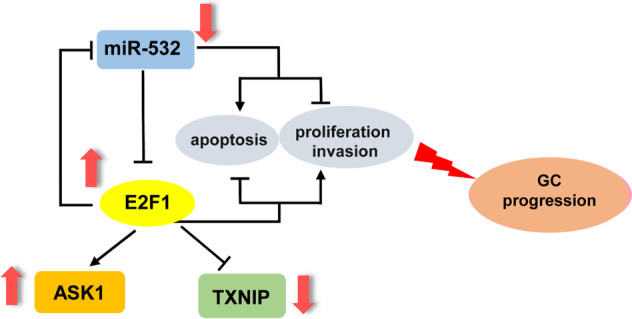
Schematic for the E2F1-miR-532 loop in GC. The miR-532-E2F1 feedback loop contributes to gastric cancer progression.

E2F1 is a representative transcription factor that plays vital roles in tumor progression [2, 37]. In this study, we showed that E2F1 act as a tumor promoter in GC progression and correlate with poor prognosis of GC patients. The functional approach of E2F1 indicated that E2F1 promoted GC cell proliferation, G1/S transition and suppressed GC cell apoptosis and DNA damage recognition foci accumulation in vitro and accelerated GC tumor growth in vivo. Furthermore, bioinformatics predictions and in vitro validation demonstrated that miR-532 can directly target E2F1 and effectively attenuate the promoting effect of E2F1 on the progression of GC in vitro and in vivo, while E2F1 can, in turn, be recruited to miR-532 promoter to inhibit the expression of miR-532. Thus, E2F1 and miR-532 form a feedback loop that may promote E2F1 and inhibit miR-532 expression in GC (Fig. 8). Although the separate role of E2F1 or miR-532 in gastric cancer has been reported in previous studies, the new E2F1-miR-532 feedback loop was first found to explain the widespread increase of E2F1 and the decrease of miR-532 in gastric cancer.

Our results may also provide new approach for GC therapies. In theory, restoring miR-532 levels in cancer would suppress oncogenic E2F1 expression, which in turn would weaken the inhibition of miR-532, thereby promoting the accumulation of miR-532 in cancer cells and delaying tumor growth. In accordance with this, restoration of miR-532 weakened the tumor-promoting effect of E2F1 in GC cells and blocked tumor growth in vivo. Therefore, E2F1 and miR-532 may be potential targets for future GC therapy. Considerable further study should be performed to make viable strategies to restore miR-532 and suppress E2F1 expression in vivo.

In summary, our study described a double-negative feedback loop comprised of E2F1 and miR-532 in gastric cancer and emphasized the critical roles of E2F1 and miR-532 in GC proliferation, G1/S transition, DNA damage and apoptosis. This result is in accordance with the idea that miRNAs form regulatory motifs with protein regulators to confer robustness to biological processes and that their disorder can expose cells to an elevated risk of dysfunction. Our findings showed that the E2F1-miR-532 loop may represent potential therapeutic approach for GC treatment.

## Supplementary information

Supplemental Figures

Original Data File

Reproducibility checklist

## Data Availability

The data used to support the findings of this study are available from the corresponding author upon request.
