# Supplementary material for: The miR-532-E2F1 feedback loop contributes to gastric cancer progression
Source: Cell Death Dis. 2022 Apr 19;13(4):376. doi: 10.1038/s41419-022-04832-7 (PMC9018701; doi:10.1038/s41419-022-04832-7)
Supplement: Supplementary file 1 — Supplemental Figures [file 41419_2022_4832_MOESM1_ESM.pdf]

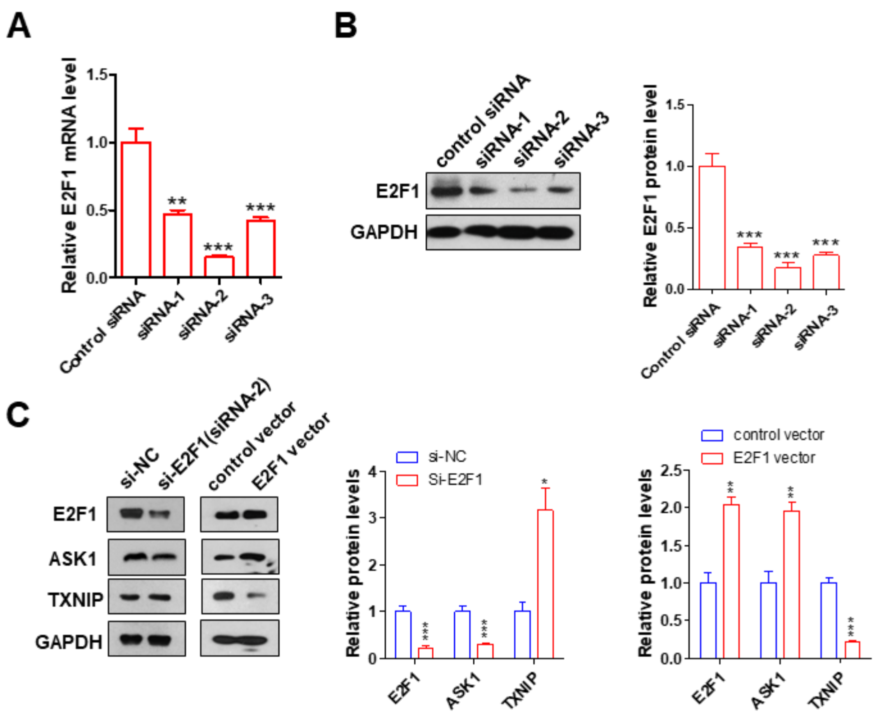

Supplementary Fig.1 The efficiencies of siRNA and overexpression vector of E2F1. (A) qRT-PCR analysis of E2F1 mRNA levels normalized to GAPDH in MKN-45 cells transfected with control siRNA, E2F1 siRNA-1, E2F1 siRNA-2 and E2F1 siRNA-3. **(B)** Western blotting analysis of E2F1 protein levels in MKN-45cells transfected with control siRNA, E2F1 siRNA-1, E2F1 siRNA-2 and E2F1 siRNA-3. GAPDH served as the loading control. **(C)** Western blotting analysis of E2F1, ASK1, TXNIP protein levels in MKN-45cells transfected with E2F1 siRNA-2 (si-E2F1) or E2F1 vector. ***$P < 0.001$; **$P < 0.01$; *$P < 0.05$.

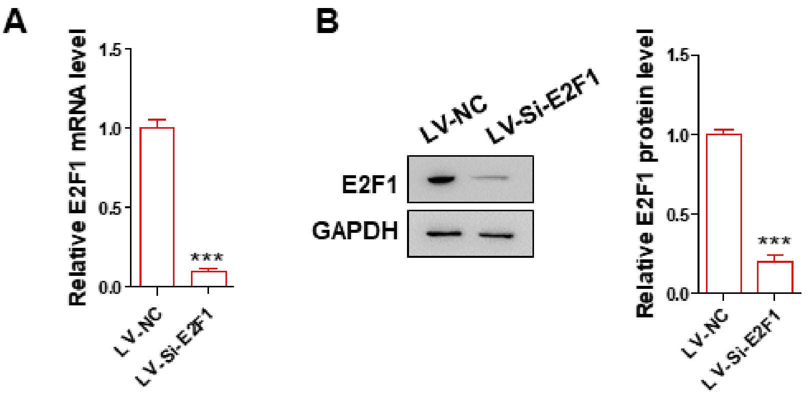

Supplementary Fig.2 The efficiencies of si-E2F1 lentivirus. (A) qRT-PCR analysis of E2F1 mRNA levels in MKN-45cells infected with control lentivirus and si-E2F1 lentivirus. **(B)** Western blotting analysis of E2F1 protein levels in MKN-45 cells infected with control lentivirus and si-E2F1 lentivirus. GAPDH served as the loading control. ***$P < 0.001$.

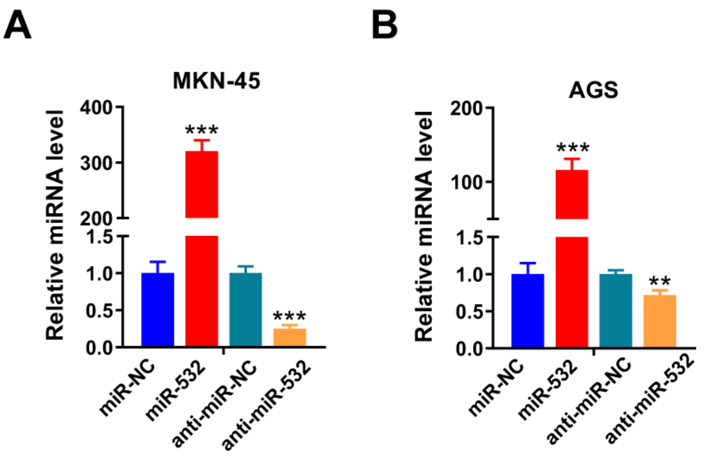

Supplementary Fig. 3 Analysis of the efficiency of miRNA mimics and inhibitors. (A, B) qRT-PCR analysis of miR-532 expression levels in MKN-45 and AGS cells after transfection with miR-532 mimics or inhibitors. U6 served as the internal reference. ***$P < 0.001$; **$P < 0.01$.

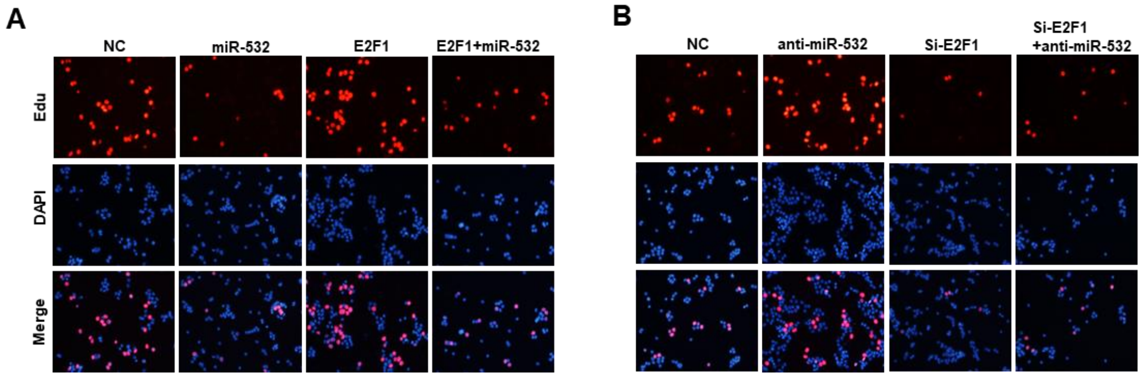

Supplementary Fig.4 The effects of miR-532 on GC cell proliferation. MKN-45 cells were transfected with NC, miR-532 mimics, E2F1 overexpressing plasmids or miR-532 mimics plus E2F1 overexpressing plasmids **(A)**, or miR-532 inhibitors, E2F1 siRNA, E2F1 siRNA plus miR-532 inhibitors**(B)**, respectively. EdU assay detected the cell proliferation.

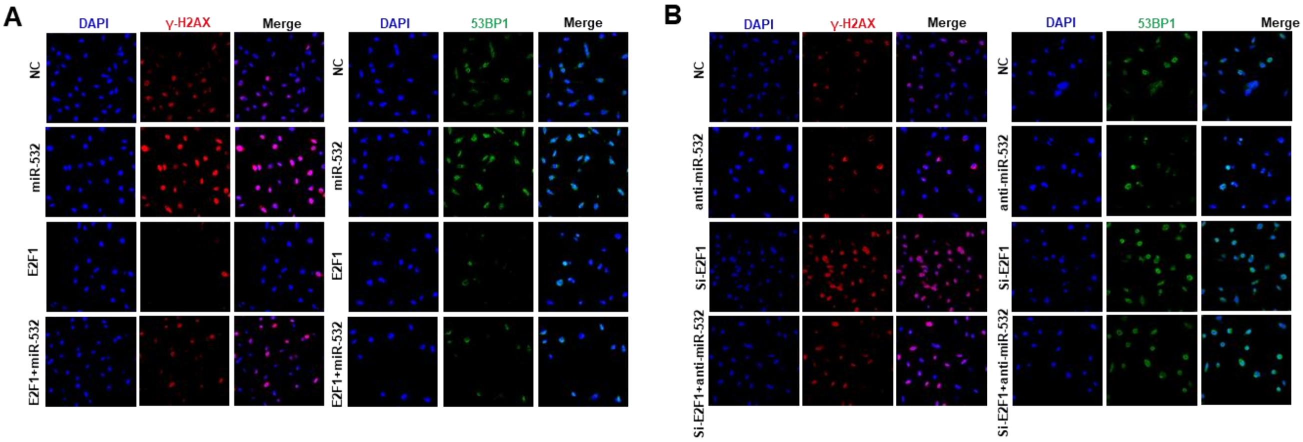

Supplementary Fig.5 MiR-532 promotes DNA damage by suppressing E2F1 in GC cells. Immunofluorescence analysis of DNA damage of MKN-45 cells transfected with NC, miR-532 mimics, E2F1 overexpressing plasmids or miR-532 mimics plus E2F1 overexpressing plasmids **(A)**, or miR-532 inhibitors, E2F1 siRNA, E2F1 siRNA plus miR-532 inhibitors**(B)**, respectively.

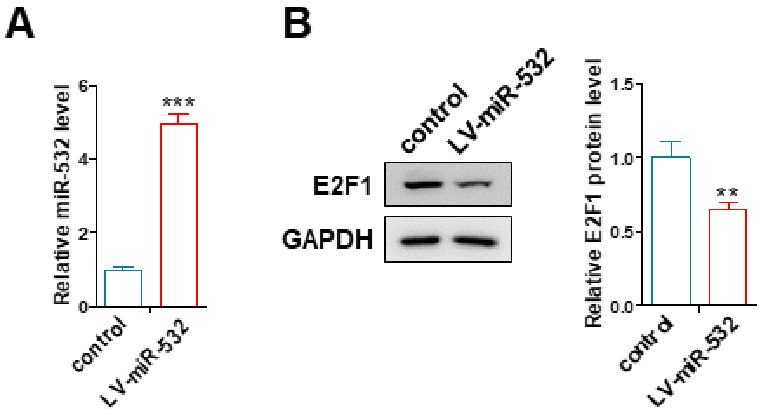

Supplementary Fig.6 Upregulation of miR-532 and downregulation of E2F1 by lentiviral expression vectors of miR-532 in MKN-45 cells. (A) qRT-PCR analysis of miR-532 expression levels in MKN-45cells infected with miR-532 lentivirus. U6 served as the internal reference. **(B)** Western blotting analysis of E2F1 protein levels in MKN-45cells infected with miR-532 lentivirus. ***$P < 0.001$; **$P < 0.01$.

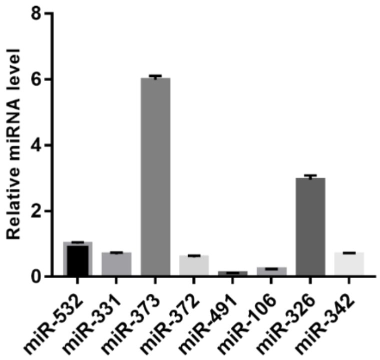

Supplementary Fig.7 RT-PCR analysis of the eight miRNAs' basic expression levels in MKN-45 cells.
