# Supplementary figures and images for: The miR-532-E2F1 feedback loop contributes to gastric cancer progression
Source: Cell Death Dis. 2022 Apr 19;13(4):376. doi: 10.1038/s41419-022-04832-7 (PMC9018701; doi:10.1038/s41419-022-04832-7)

**Fig.1**

**1-7**

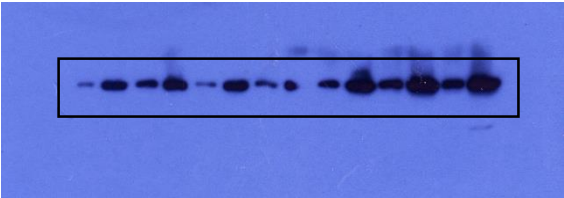

**E2F1**

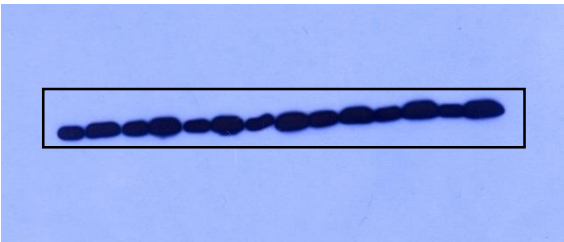

**GAPDH**

**15-21**

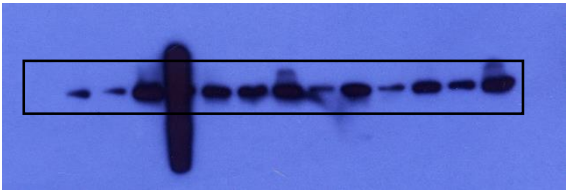

**E2F1**

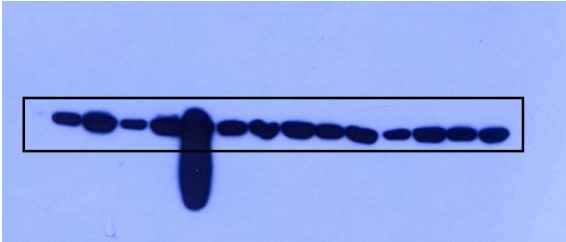

**GAPDH**

**8-14**

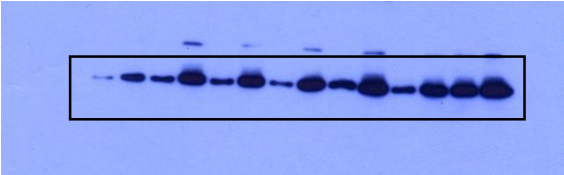

**E2F1**

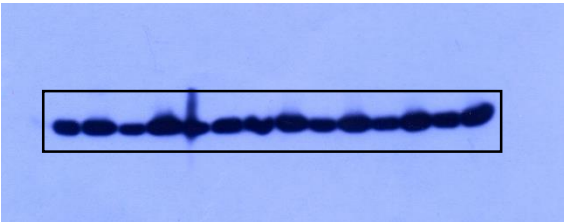

**GAPDH**

**Fig.2**

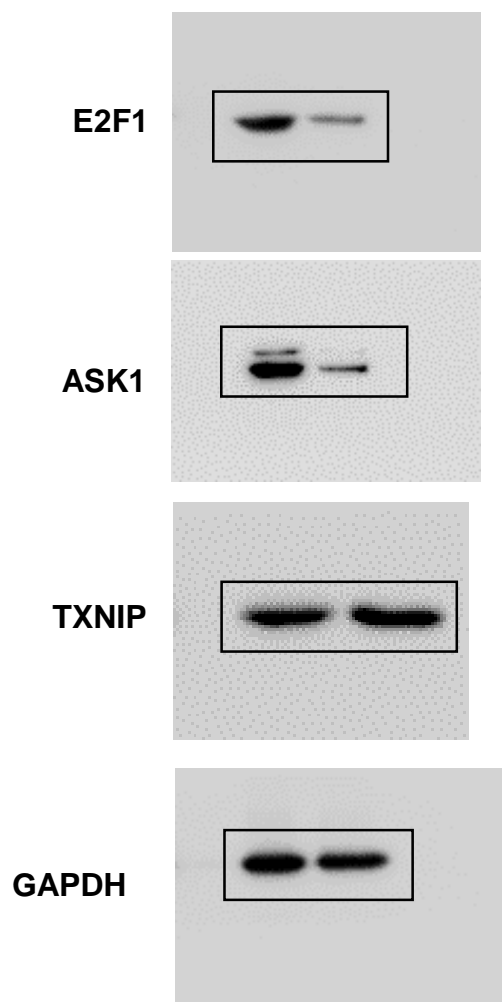

**Fig.4**

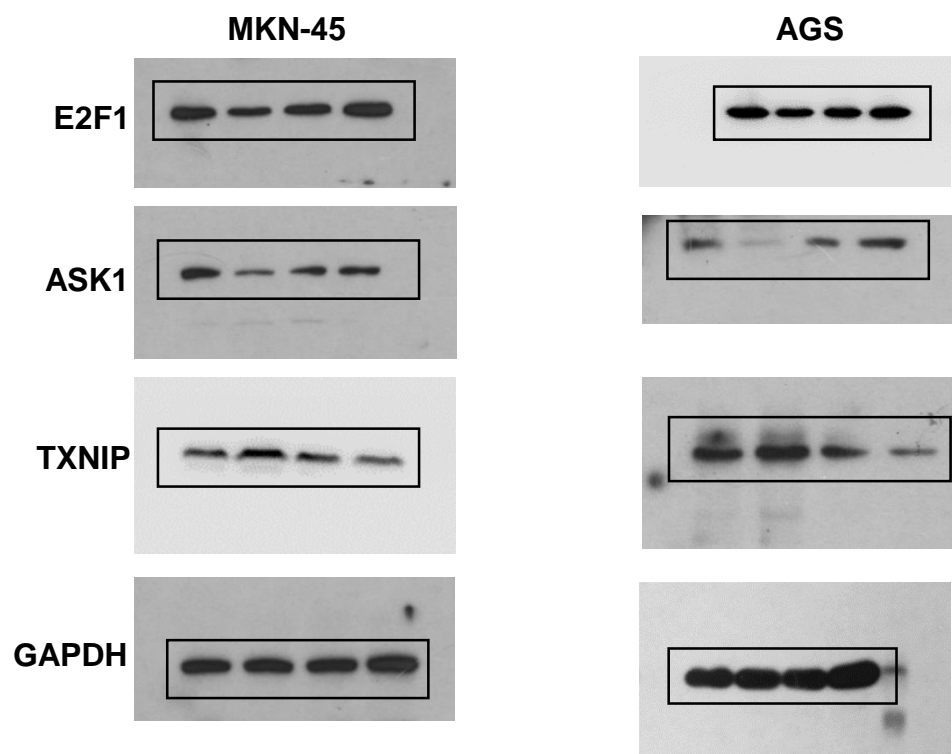

**Fig.4**

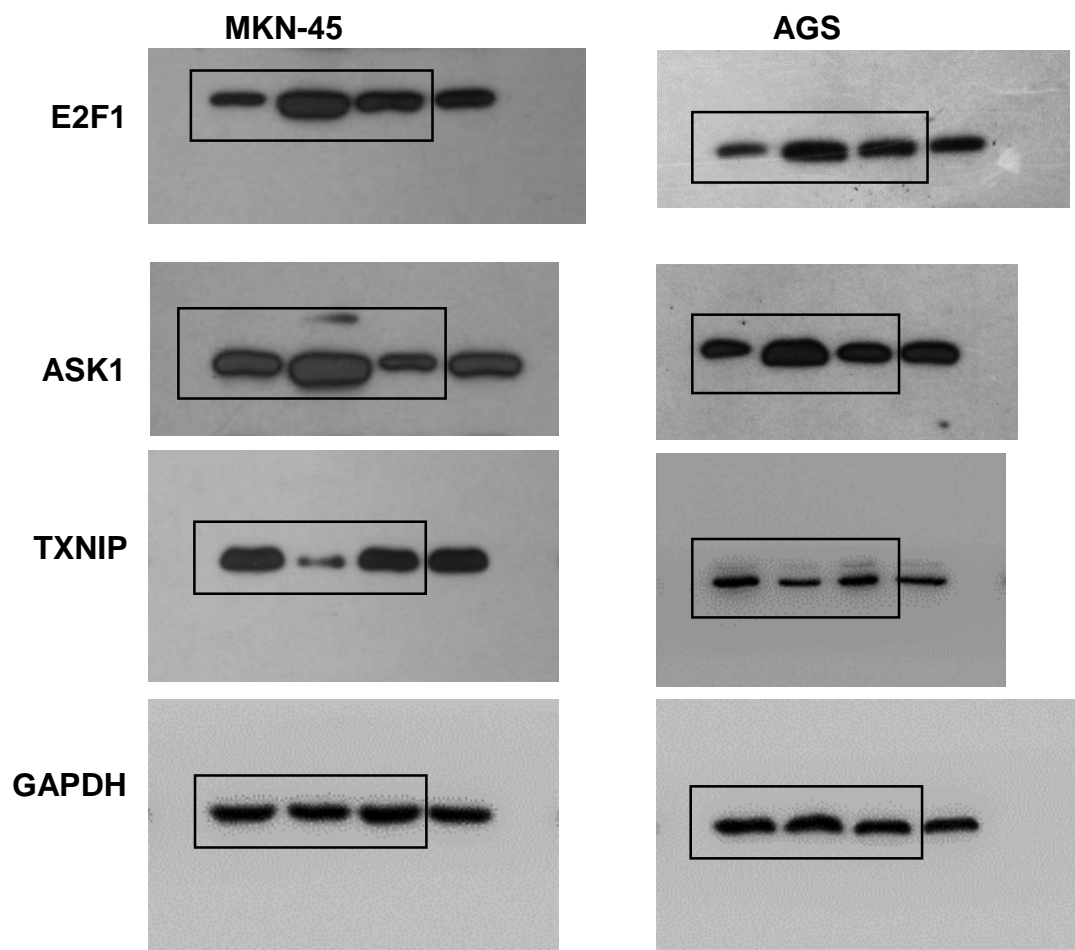

Fig.6

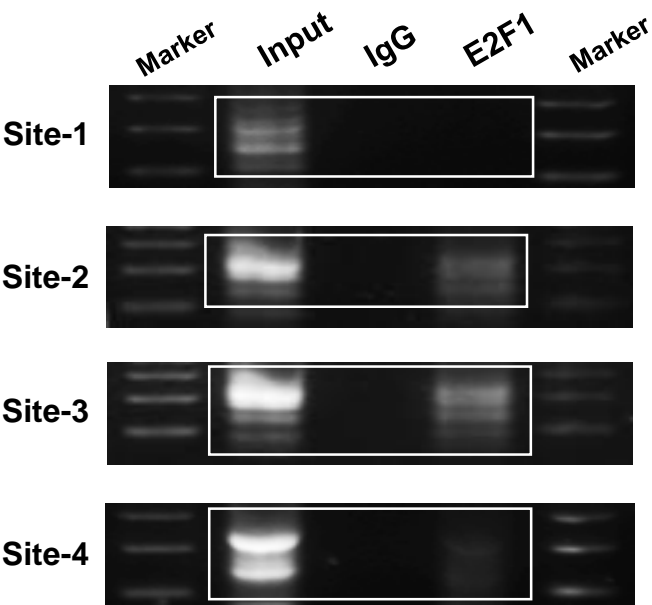

**Fig.7**

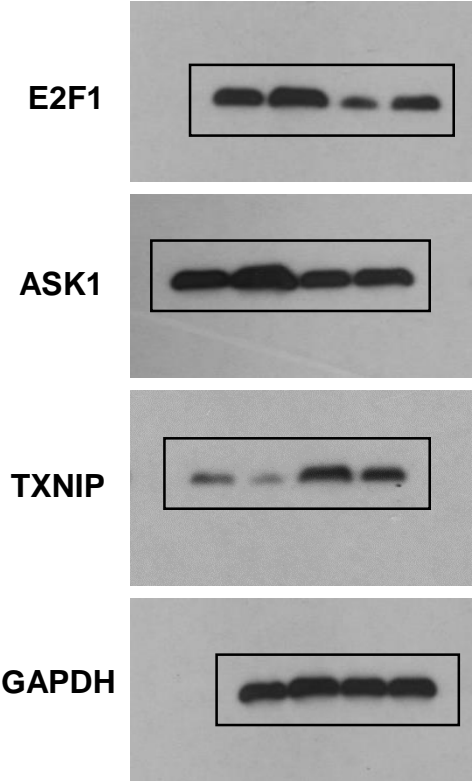

**sFig.1**

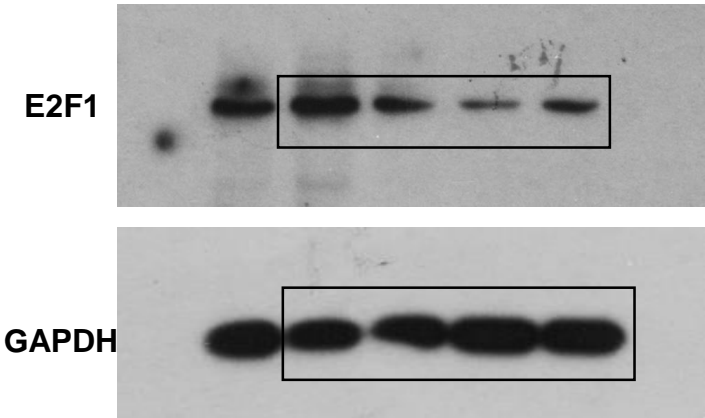

**sFig.1**

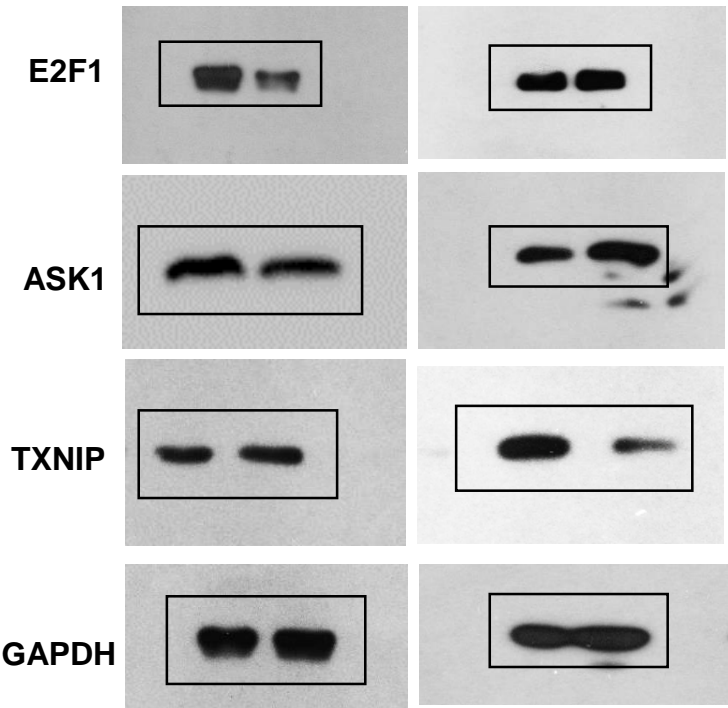

**sFig.2**

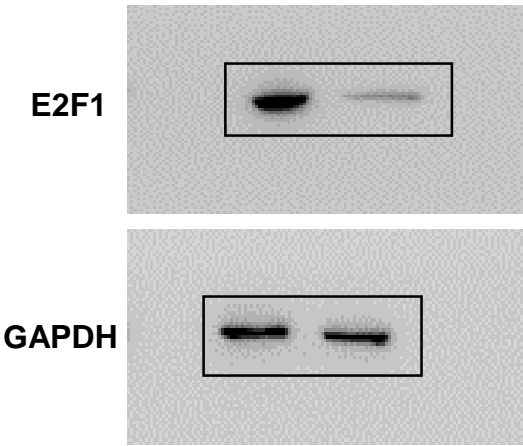

**sFig.6**

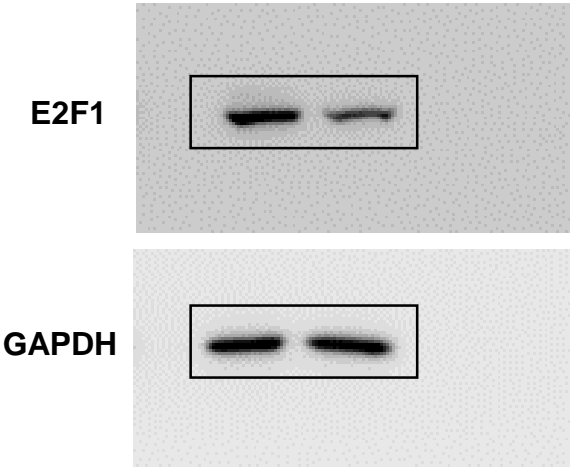

Supplement: Supplementary file 2 — Original Data File [file 41419_2022_4832_MOESM2_ESM.pdf]
